# Biological Properties of Extracts Obtained from In Vitro Culture of *Plectranthus scutellarioides* in a Cell Model

**DOI:** 10.3390/ijms25021043

**Published:** 2024-01-15

**Authors:** Tomasz Kowalczyk, Joanna Sikora, Anna Merecz-Sadowska, Wirginia Kukula-Koch, Ewelina Synowiec, Agata Majda, Dawid Juda, Tomasz Śliwiński, Przemysław Sitarek

**Affiliations:** 1 Department of Molecular Biotechnology and Genetics, Faculty of Biology and Environmental Protection, University of Lodz, Banacha 12/16, 90-237 Lodz, Poland; 2 Department of Bioinorganic Chemistry, Medical University of Lodz, Muszynskiego 1, 90-151 Lodz, Poland; joanna.sikora@umed.lodz.pl; 3 Department of Economic and Medical Informatics, University of Lodz, 90-214 Lodz, Poland; anna.merecz-sadowska@uni.lodz.pl; 4 Department of Allergology and Respiratory Rehabilitation, Medical University of Lodz, 90-725 Lodz, Poland; 5 Department of Pharmacognosy with Medicinal Plants Garden, Medical University of Lublin, 1 Chodzki Str., 20-093 Lublin, Poland; virginia.kukula@gmail.com; 6 Laboratory of Medical Genetics, Faculty of Biology and Environmental Protection, University of Lodz, Pomorska 141/143, 90-236 Lodz, Poland; ewelina.synowiec@biol.uni.lodz.pl (E.S.); tomasz.sliwinski@biol.uni.lodz.pl (T.Ś.); 7 Students Research Group, Department of Medical Biology, Medical University of Lodz, 90-151 Lodz, Poland; agata.majda@stud.umed.lodz.pl (A.M.); dawid.juda@stud.umed.lodz.pl (D.J.); 8 Department of Medical Biology, Medical University of Lodz, Muszynskiego 1, 90-151 Lodz, Poland

**Keywords:** in vitro plant culture of *Plectranthus scutellarioides* extracts, phytochemical screening, cytotoxic effect, apoptosis induction, antiplatelet effect

## Abstract

*Plectranthus scutellarioides* (L.) R.Br. is a medicinal plant that has long been used in traditional medicine to treat conditions such as abscesses, ulcers, and ear and eye infections. It is known to have a wide range of biological properties, such as antibacterial, antioxidant, antifungal, anti-inflammatory, anti-diabetic and anti-cancer effects. In this study, we established in vitro cultures from both the aerial parts and roots of *Plectranthus scutellarioides*. Subsequently, we compared the basic phytochemical profile of the obtained extracts and conducted a biological analysis to assess their potential for inducing apoptosis in breast (MCF-7) and lung (A549) cancer cells. Phytochemical analysis by HPLC-MS revealed the presence of compounds belonging to phenolic acids (ferulic, syringic, vanillic, rosmarinic, chlorogenic, caffeic, coumaric, dihydroxybenzoic acids), flavonoids (eriodyctiol and cirsimaritin), and terpenes such as 6,11,12,14,16-Pentahydroxy-3,17diacetyl-8,11,13-abietatrien-7-one, 6,11,12,14,16-Pentahydroxy-3,17-diacetyl5,8,11,13-abietatetraen-7-one, and 3,6,12-Trihydroxy-2-acetyl-8,12-abietadien7,11,14-trione. The results show that both extracts have a cytotoxic and genotoxic effect against MCF-7 and A549 cancer cells, with a different degree of sensitivity. It was also shown that both extracts can induce apoptosis by altering the expression of apoptotic genes (*Bax*, *Bcl-2*, *TP53*, *Fas,* and *TNFSF10*), reducing mitochondrial membrane potential, increasing ROS levels, and increasing DNA damage. In addition, it has been shown that the tested extracts can alter blood coagulation parameters. Our results indicate that extracts from in vitro cultures of *Plectranthus scutellarioides* aerial parts and roots have promising therapeutic application, but further research is needed to better understand the mechanisms of their action in the in vitro model.

## 1. Introduction

Plants have been used since ancient times to treat ailments and diseases faced by humans. They are an inexhaustible source of valuable secondary metabolites, and their extracts, as well as pure compounds isolated from their various parts, exhibit a range of biological properties with multidirectional effects [1,2,3]. The genus *Plectranthus* is an especially interesting group of plants, with notable biological properties such as antibacterial, antioxidant, antifungal, anti-inflammatory, anti-diabetic, and anti-cancer effects [4,5]. One species with potential pharmacological activity is *Plectranthus scutellarioides* (L.) R.Br (syn. *Coleus scutellarioides*, *Coleus blumei*, *Plectranthus blumei*), a member of the Lamiaceae family, which has more than 173 varieties worldwide and is found in tropical Africa, Asia, and Australia [6,7,8]. Thanks to its valuable secondary metabolites, such as terpenoids, flavonoids, phenolic compounds, alkaloids, saponins, tannins, etc. [8], it is used in folk medicine by the Mexican people as an infusion to treat gastrointestinal diseases or as a hallucinogen in the Oaxaca region [9]. In the past, it was used by the Lohit people of India to treat people who had been stung by a scorpion [10]. In Indonesia, however, *P. scutellarioides* is used fresh, boiled, or infused to treat conditions such as abscesses, ulcers, and ear and eye infections. Its root is used to cure diarrhea, abdominal pain, diabetes, constipation, fever, and menstrual pain [11,12]. The leaves are used to treat pulmonary tuberculosis by the Toraja ethnic group in Indonesia. This shows the importance of this plant and its pharmacological potential [13]. Hanum et al. showed that *P. scutellarioides* leaf extract had antibacterial activity against Escherichia coli and Staphylococcus aureus bacteria [14]. In turn, Fakhriati et al. found that *P. scutellarioides* extract was the most potent anti-inflammatory agent, inhibiting nitrite production by macrophages [15]. Due to the rather limited number of studies on this species, it is critical to learn about its new biological properties.

Micropropagation is one of the fields of plant biotechnology that allows plant parts to be manipulated and obtained in vitro under controlled conditions on appropriately selected substrates. In this way, it is possible to obtain maternal and reproductive material that is free of any diseases and pests. In addition, this method allows new varieties to be quickly introduced to the market and, also importantly, allows a large amount of material to be obtained in a short period of time without degrading the environment, which is crucial in light of a widely understood ecology. In this work, plants grown in vitro were used to check the biological properties of their extracts for further potential development of an efficient source of valuable biologically active compounds. It is also known that in some cases the accumulation of metabolites in vitro may exceed their content in naturally growing plants. Moreover, the possibility of precise use of elicitation may allow the creation of an efficient system for the production of biologically active plant compounds [16,17,18]. Previous studies show that the species *P. scutellarioides* has been introduced into in vitro culture by micropropagation through manipulation of various medium components and growth hormones [19,20,21], but this study represents the first time that the material obtained has been subjected to bioassays in an in vitro model.

The aim of this study was to obtain in vitro cultures of aerial parts (AEPS) and roots (REPS) of *Plectranthus scutellarioides* and then, from the extracts obtained, to compare their preliminary phytochemical screening, to check the cytotoxic effect and the induction of apoptosis in breast (MCF-7) and lung (A549) cancer cell lines by altering apoptotic gene expression, mitochondrial potential, ROS, genotoxic effects, and the effect on blood coagulation parameters.

## 2. Results

### 2.1. Aerial Parts and Roots of Plectranthus scutellarioides in In Vitro Culture

**Figure 1:**
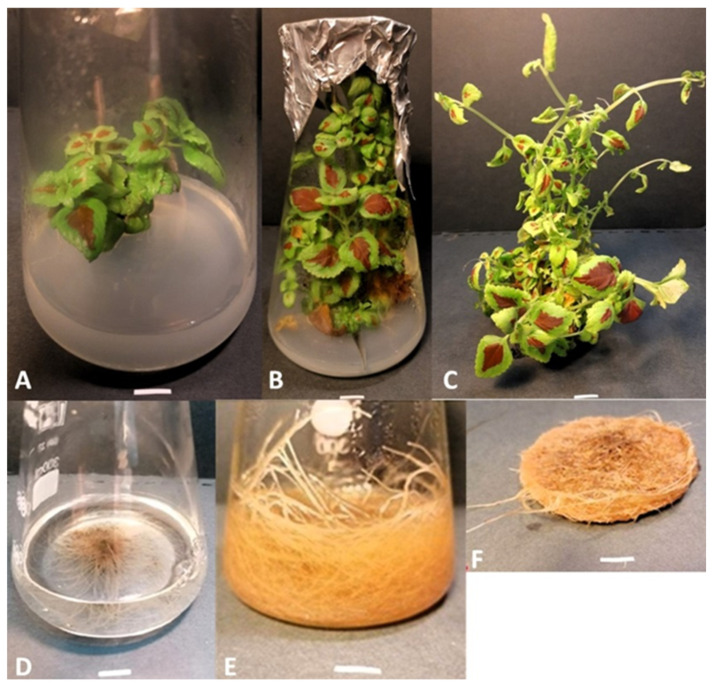
*P. scutellarioides* plant grown in vitro: (**A**) two-week-old growth, (**B**,**C**) five-week-old growth, (**D**) roots of *P. scutellarioides* grown in vitro (culture initiation), (**E**,**F**) four-week-old root culture obtained in vitro. Bar = 1 cm.

AEPS and REPS were used to prepare the extract. Roots were cultured in liquid SH medium containing 0.2 mg/L indole-3-butyric acid (IBA). Plants, on the other hand, were grown on solid MS medium with 0.7% agar containing 3% sucrose and 1 mg/L 6-benzylaminopurine (BAP). The in vitro cultures that were used to prepare the extracts are shown in Figure 1.

### 2.2. Preliminary Phytochemical Screening of the Aerial Parts and Roots of Plectranthus scutellarioides Extracts

**Figure 2:**
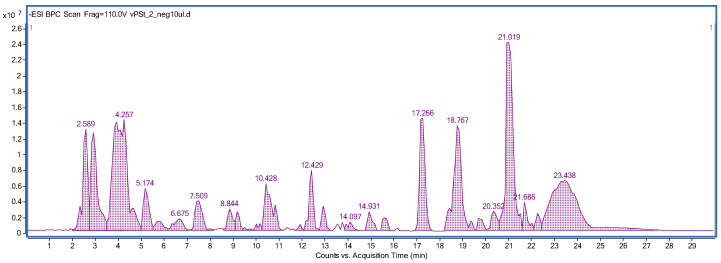
The mass chromatogram of the aerial parts recorded in the negative ionization mode.

The applied chromatographic methodology provided clear mass chromatograms (see Table S1 in the Supplementary File). The negative ionization mode was found preferential in the identification of polyphenols in the tested extract (see Figure 2 and Table 1). The HPLC-MS fingerprinting of the analyzed AEPS and REPS revealed the presence of two major groups of natural products in the analyzed samples. Among them, polyphenols were the leading components, comprising various representatives from the group of phenolic acids, which included ferulic, syringic, vanillic, rosmarinic, chlorogenic, caffeic, coumaric, and dihydroxybenzoic acids. Among the flavonoids, eriodyctiol and cirsimaritin were identified. Also, next to the aforementioned metabolites, interesting terpene compounds were identified in the samples. The 6,11,12,14,16-pentahydroxy-3,17-diacetyl-8,11,13-abietatrien-7-one, 6,11,12,14,16-pentahydroxy-3,17-diacetyl-5,8,11,13-abietatetraen-7-one, and 3,6,12-trihydroxy-2-acetyl-8,12-abietadien-7,11,14-trione represented the group of triterpenes. Certainly, the most important metabolite that was detected in the sample was rosmarinic acid, with its prominent peak in both tested extracts. Another molecule with a similar molecular formula was detected in the sample that differed from the rosmarinic acid, with the intensity of single *m*/*z* fragments visible in the MS/MS spectrum (see Table S2 in the Supplementary File). The other analogue is possibly composed of the same units, attached in a different position of the unsaturated ring. The presence of rosmarinic acid could show an impact on the total activity of the extracts. The identified compounds are listed in Table 1, and their MS/MS spectra are presented in the supplementary file (Table S2). The qualitative fingerprint of the extracts obtained from the roots and overground parts of the plant were alike; however, the concentrations of single molecules in both organs differed markedly. Table 1 presents the differences in the peak areas recorded for the tentatively identified components. In general, the overground parts of the plant were found to be better sources of a few metabolites: rosmarinic acid, coumaric acid, and rosmarinic acid isomer. Also, the peak area of cirsmaritin and ferulic acid were three times higher in the above-ground parts. Syringic acid was present in a 10-fold smaller concentration in the roots than in the green parts of the plant.

**Figure 3:**
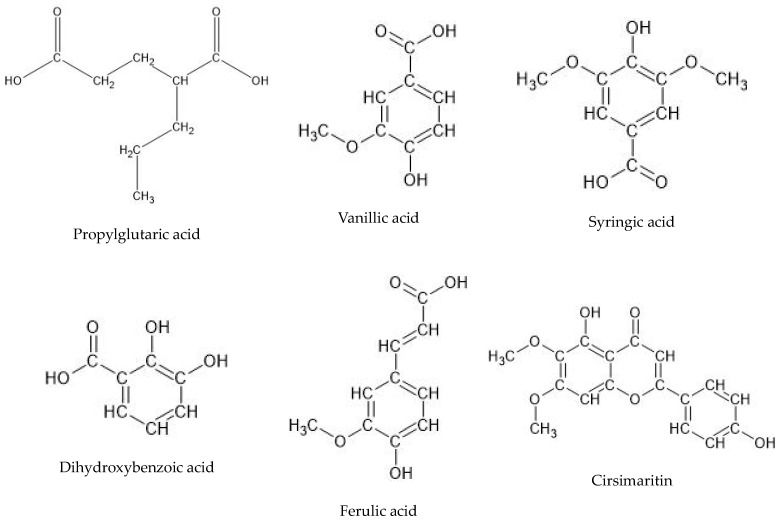
Selected chemical structures of key phytocompounds identified in *P. scutellarioides* extracts.

Key bioactive phytochemical constituent structures identified within the *P. scutellarioides* extracts (+++ for aerial parts and roots) are presented in Figure 3.

### 2.3. The Cytotoxic Effect of the Aerial Parts and Roots of Plectranthus scutellarioides Extracts on Breast Cancer Cells (MCF-7), Human Lung Adenocarcinoma Cells (A549) and Normal Human Gingival Fibroblasts (HGF-1)

The cytotoxic effects after 24 h showed that AEPS significantly decreased MCF-7 cancer cell survival to 50% (IC_50_) at a concentration of 0.125 mg/mL. In contrast, REPS exhibited weaker cytotoxicity, with an IC_50_ of 0.726 mg/mL. For the A549 cell line, REPS demonstrated increased potency, reducing cell viability by 50% at a concentration of approximately 0.250 mg/mL, while AEPS had an IC_50_ value of 0.850 mg/mL (Figure 3).

**Figure 4:**
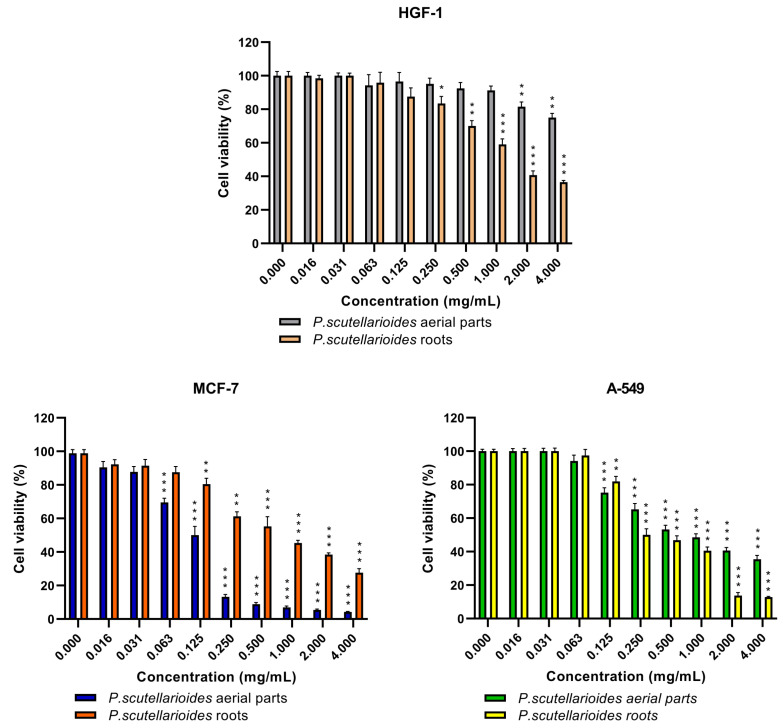
Cell viability of normal HGF-1 as well as MCF-7 and A549 cancer cells after treatment with AEPS and REPS for 24 h. * *p* < 0.05, ** *p* < 0.01, *** *p* < 0.001—untreated cells vs. cells treated with the extracts after 24 h.

On normal HGF-1 cells, REPS elicited a statistically significant decline in cell viability at concentrations of 0.250 mg/mL and higher. Meanwhile, AEPS did not cause substantial cytotoxic effects up to 2 mg/mL, with HGF-1 survival diminishing significantly at higher concentrations. It is noteworthy, however, that despite this, the IC_50_ value for the cancer cell line was not toxic to the normal line and caused a decrease in survival (Figure 4).

### 2.4. Morphological Observations under Microscopy after Treatment with the Aerial Part and Root of Plectranthus scutellarioides 

**Figure 5:**
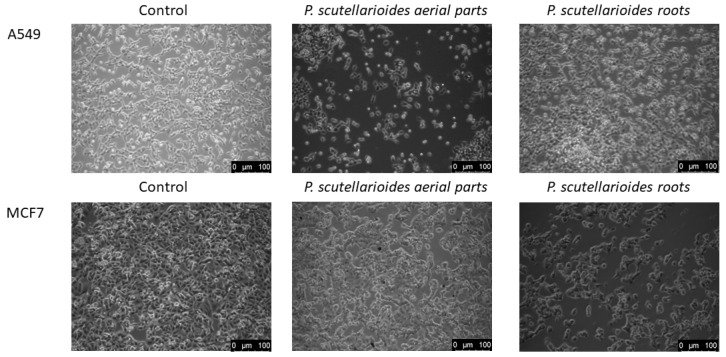
Microscope images of A549 and MCF-7 cells after 24 h incubation in IC_50_ concentration with AEPS and REPS vs. cells from untreated culture.

MCF-7 and A549 cancer cells showed morphological changes characteristic of the apoptosis process (cell shrinkage, blebbing on the cell membrane, and loss of contact with neighboring cells) after 24 h incubation with AEPS and REPS at IC_50_ compared to untreated cells (Figure 5).

### 2.5. Apoptotic Genes Expressed following Treatment with Plectranthus scutellarioides Aerial and Root Extracts

The results of RT-PCR analysis showed a change in the expression of the genes tested (*Fas*, *TNFSF10*, *TP53*, *Bax*, *Bcl-2*) in MCF-7 and A549 cancer cells after 24 h incubation with AEPS and REPS at IC_50_ concentration.

**Figure 6:**
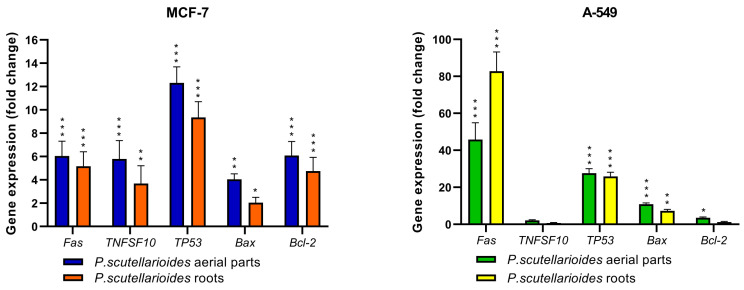
Expression of apoptotic genes after 24 h exposure to AEPS and REPS at IC_50_ concentration. Data are presented as fold change in cells treated with AEPS and REPS to untreated cells, for which the expression level was taken as 1. Mean values ± SD (*n* = 3). * *p* < 0.05, ** *p* < 0.01, *** *p* < 0.001.

For the MCF-7 cancer cell line, an increase in the expression of apoptotic genes (*Bax*, *Bcl-2*, *Fas*, *TP53*, *TNFSF10*) was shown for both extracts tested, although the highest expression was observed for the *TP53* gene. Specifically, AEPS elevated TP53 expression 12.31-fold compared to untreated control cells, while REPS caused a 9.36-fold upregulation. In contrast, line A549 showed a significant increase in expression for the *Fas* gene after treatment with AEPS and REPS, of 45.75-fold and 82.77-fold, respectively, compared to the control. An increase in expression levels was also shown for the *TP53* and *Bax* genes for both extracts, while these changes were not observed for the *TNFSF10* gene for both extracts and the *Bcl-2* gene for REPS (Figure 6).

### 2.6. Measurement of ROS Generation

The results showed that the production of ROS was increased in MCF-7 and A549 cells exposed to AEPS and REPS at an IC_50_ concentration after 1 h and after 24 h compared to the control.

**Figure 7:**
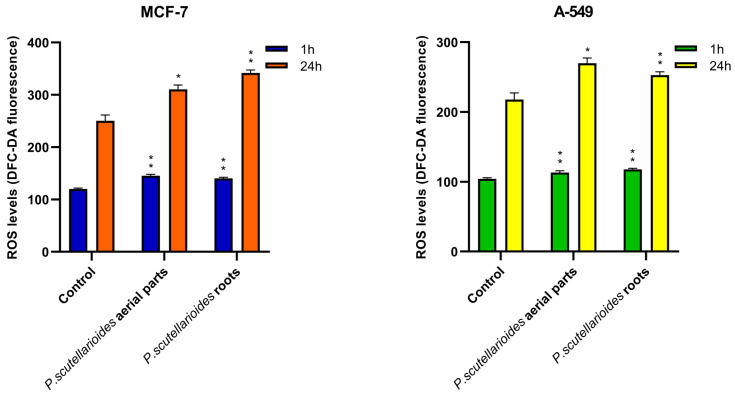
ROS generation in MCF-7 and A549 cells treated with IC_50_ concentrations of AEPS and REPS for 1 h and 24 h. Results are given as means ± SD. * *p* < 0.05, ** *p* < 0.01, significant differences between treated and untreated (control) cells.

After 1 h, ROS levels were enhanced 1.21-fold (MCF-7) and 1.09-fold (A549) following treatment with AEPS, and 1.17-fold (MCF-7) and 1.13-fold (A549) with REPS as compared to the control. Prolonged 24 h incubation resulted in further ROS stimulation, with AEPS-mediated elevations of 1.24-fold (MCF-7) and 1.70-fold (A549), and REPS showing ROS increases of 1.36-fold (MCF-7) and 1.16-fold (A549) (Figure 7).

### 2.7. Measurement of Mitochondrial Membrane Potential (MMP)

**Figure 8:**
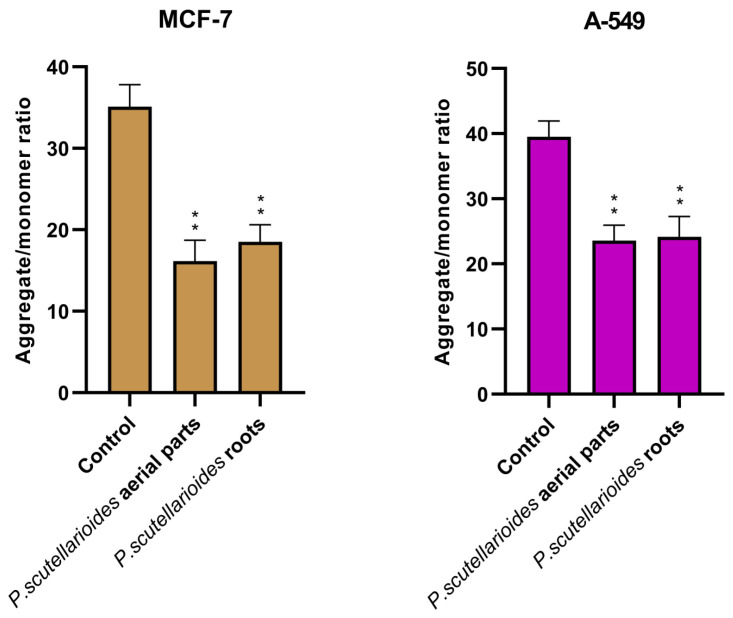
Changes in the mitochondrial membrane potential of MCF-7 and A549 cells treated with IC_50_ concentrations of AEPS and REPS and incubated for 24 h. The results are expressed as the mean ± SD. ** *p* < 0.01, significant differences between treated and untreated (control) cells.

Quantification of MMP changes verified apoptotic pathway engagement in both cell lines by AEPS and REPS. After 24 h exposure, AEPS reduced MMP levels by 45.97% in MCF-7 and 59.62% in A549 cells compared to the control. Meanwhile, REPS decreased potentials by 52.72% (MCF-7) and 61.06% (A549) (Figure 8).

### 2.8. Measurement of DNA Double Strand Breaks by Comet Assay

**Figure 9:**
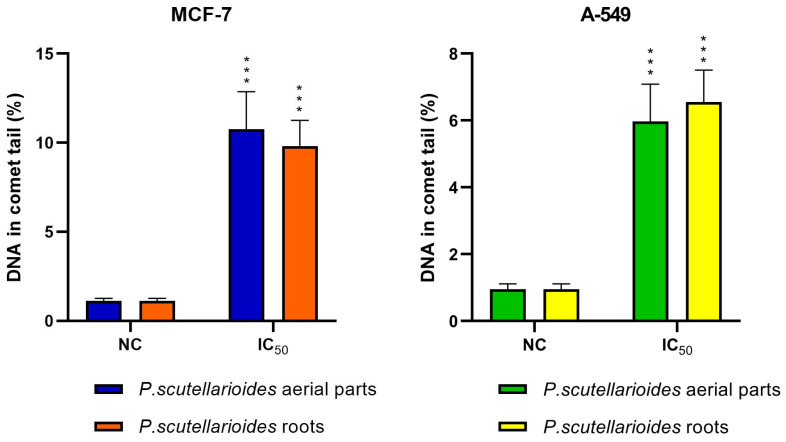
DNA damage in MCF-7 and A549 cells after treatment with AEPS and REPS after 24 h. Data are expressed as means ± SD of three independent experiments. NC—negative control, *** *p* < 0.001—untreated cells vs. cells treated with the extracts after 24 h.

The effect of AEPS and REPS on DNA damage in MCF-7 and A549 cells was tested using the comet assay. Both plant extracts at IC_50_ concentrations caused statistically significant DNA damage in MCF-7 and A549 cells in comparison with the control. At 0.125 mg/mL, AEPS increased MCF-7 DNA tail fractions 9.60-fold, while at 0.850 mg/mL, it raising A549 fractions 6.28-fold compared to the control. Similarly, 0.726 mg/mL and 0.250 mg/mL REPS boosted MCF-7 and A549 tail fractions approximately 8.77-fold and 6.88-fold, respectively (Figure 9).

### 2.9. Biocompatibility Studies

**Figure 10:**
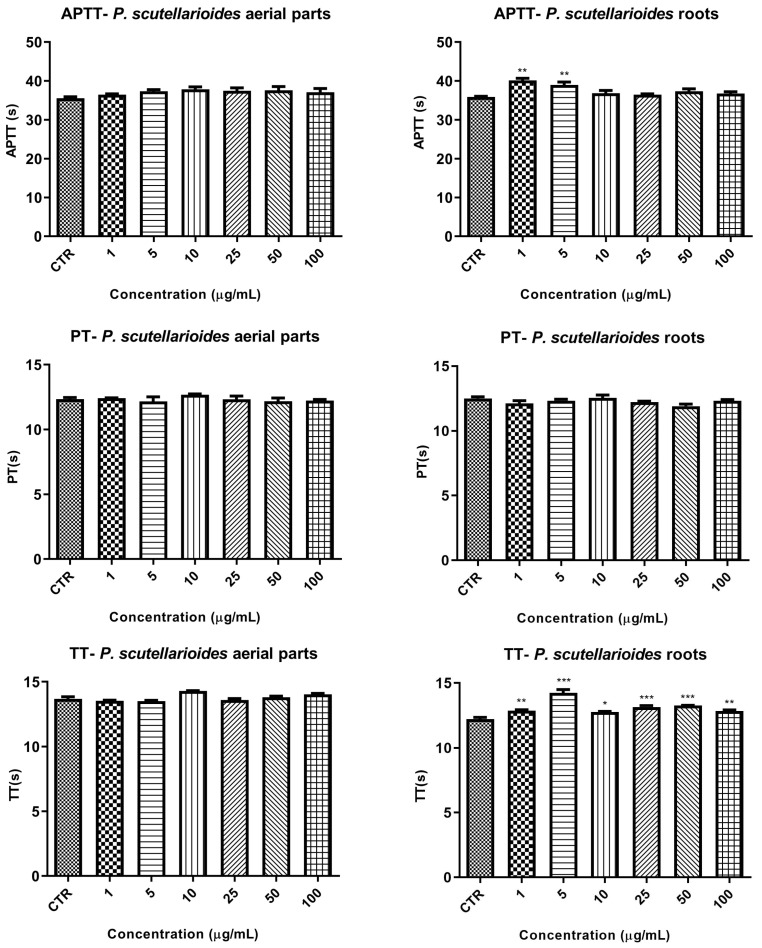
Effects of plasma incubation with standard concentration (range 1–100 µg/mL) of AEPS and REPS on basic coagulation parameters: activated partial thromboplastin time (APTT), prothrombin time (PT), and thrombin time (TT). The reference values for each test are as follows: PT: 9.7–14.6 s; APTT: 26.7–40.0 s; TT: 11.0–15.0 s The results are presented as mean ± S.D.; *n* = 4 (in duplicate). Values are statistically significant in comparison with control samples. * *p* < 0.05, ** *p* < 0.01, *** *p* < 0.001.

A preliminary study of biocompatibility using human blood incubated with various concentrations (range 1–100 µg/mL) of AEPS and REPS was performed. First, the impact of the extracts on the plasma coagulation process was assessed. For this purpose, three routine laboratory tests (Prothrombin Time—PT, Activated Partial Thromboplastin Time—APTT, Thrombin Time—TT) were carried out. As shown in Figure 10, the incubation of plasma with AEPS did not have a statistically significant effect on the coagulation process. After incubation with REPS, a mild increase in APTT was observed at low concentrations (1 and 5 µg/mL) and in TT over the entire concentration range tested. In none of the examined cases were the values outside the reference range.

### 2.10. The Effect of Plectranthus scutellarioides Aerial Part and Root Extracts on RBC Hemolysis

**Figure 11:**
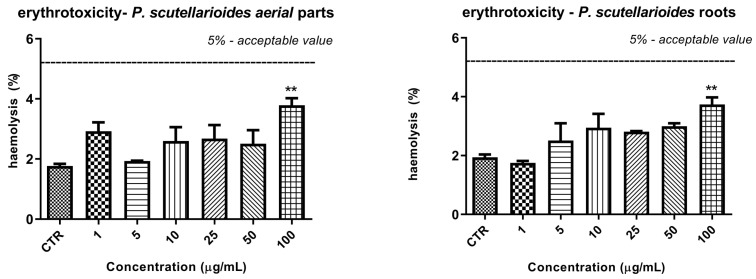
Percentage of hemolysis obtained from the interaction of standard concentrations (range 1–100 µg/mL) of *P. scutellarioides* roots and *P. scutellarioides* aerial extracts with 2% red blood cell suspension. The results are presented as mean ± S.D.; *n* = 3, ** *p* < 0.01 vs. control.

The effect of both extracts on erythrocyte hemolysis is shown in Figure 11. In this test, no statistically significant effect on erythrocyte membrane integrity was observed. A statistically significant increase in % RBC hemolysis was observed only at the highest concentration (100 µg/mL) of both extracts. In the case of REPS, this was 3.74 ± 0.41% compared with 1.94 ± 0.17% in the control sample, while in the case of AEPS it was 3.80 ± 0.41% compared with 1.77 V 0.12% in the control sample. However, the value of 5%, which is considered to be a safe level of hemolysis, was not exceeded in any case.

### 2.11. Microscopic Observations after the Effect of Plectranthus scutellarioides Aerial Part and P. scutellarioides Root Extracts on RBCs

**Figure 12:**
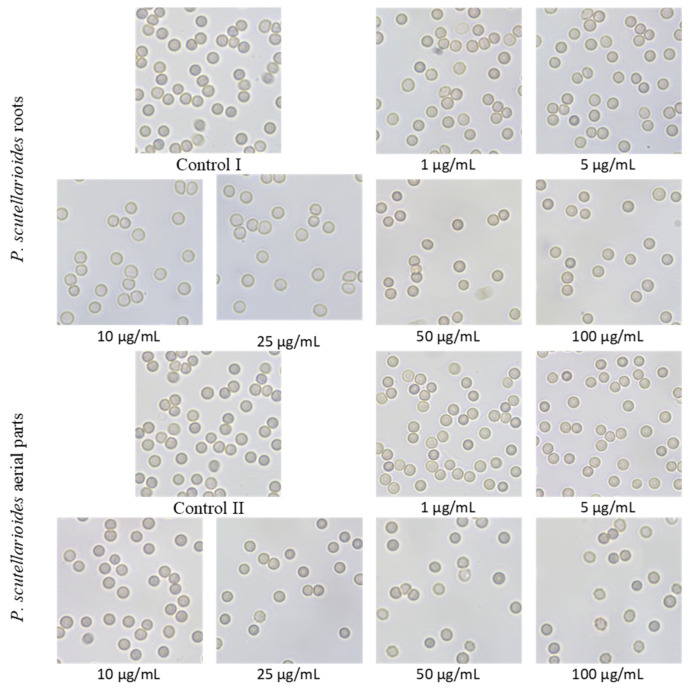
Effects of AEPS and REPS on the morphology of red blood cells in vitro after 24 h incubation at 37 °C. Representative phase-contrast images are shown (magnification = 400 times).

The absence of a significant effect of both extracts on the erythrocytes was also confirmed by microscopic studies of the morphology of these cells. No pathological forms were observed over the whole range of concentrations tested, as shown in Figure 12. Individual echinocytes observed at concentrations above 25 µg/mL of *P. scutellarioides* aerial extract are the physiological and reversible forms.

### 2.12. Effects of Plectranthus scutellarioides Root and Plectranthus scutellarioides Aerial Part Extracts on Parameters of White Thrombus Formation Process

The last study in the field of biocompatibility was the evaluation of the extracts’ impact on blood platelet activation and the formation of white clots under simulated blood flow conditions. In this case as well, no significant influence on the parameters assessing this process (Occlusion Start Time—OST, Occlusion Time—OT, Area Under Curve—AUC) was observed. The study was conducted on four healthy volunteers with low baseline risk of adverse events in hemostasis, and this risk remained unchanged after incubation with both of the tested extracts (Table 2).

## 3. Discussion

Plants are inexhaustible sources of many bioactive substances and have been used for years to treat various diseases. The importance of plants as therapeutic ingredients is increasingly recognized with current technological advances. Their secondary metabolites often exhibit a broad spectrum of biological properties, such as anticancer, anti-inflammatory, antimicrobial, antioxidant, or antiviral effects. Furthermore, many compounds used in cancer therapies are of plant origin. Traditional and modern medicine offer promising methods for the discovery and commercialization of new phytochemicals [22,23,24,25,26]. In addition to traditional methods, the extraction of valuable secondary metabolites can be achieved through biotechnology, i.e., plant in vitro cultures and micro-propagation, which is an alternative to wild species and allows different material to be obtained in vitro on a larger scale and, most importantly, without destroying the environment [27,28].

The aim of the study was the establishment of in vitro cultures of aerial parts and roots of *P. scutellarioides*, followed by a preliminary phytochemical analysis of the material obtained and the testing of biological properties such as cytotoxic effect in MCF-7 breast and A549 lung cancer cell lines and induction of apoptosis by alteration of apoptotic gene expression, change in mitochondrial potential, change in ROS levels, genotoxic effect, and the effect of the extract on selected blood biochemical parameters. The first step in the study was to obtain in vitro material from the aerial parts and roots of *P. scutellarioides* by micropropagation. Explants were cultured on MS media with agar and 1 mg/mL BAP for aerial parts. Root culture, on the other hand, was carried out on SH liquid medium with 0.2 mg/mL IBA. These were the optimal growth conditions for our cultures and allowed us to obtain a considerable amount of material necessary for further experiments. In an earlier study by Rani et al., it was found that MS medium with 2 mg/mL BA and 1 mg/mL NAA was the most optimal for the growth of aerial parts, while for roots the best results were obtained with MS medium with 2 mg/mL IBA, which is in agreement with our results [29]. In another study, Bauer et al. showed that roots were regenerated on young leaf explants grown on MS medium supplemented with 1 mg/L NAA [30]. In contrast, Sahu et al. obtained in vitro shoot cultures on MS medium supplemented with BAP at a concentration of 0.5 mg/L [19]. Marcotrigiano et al. showed an increase in shoot culture on MS medium with the addition of 1 to 3 mg BA mg/L, which is consistent with previous studies [31]. All the examples presented confirm stable growth cultures (aerial parts and roots) that can be the starting point for further analysis.

The next step was to make the extracts from the collected material in vitro from the aerial parts and roots of *P. scutellarioides* and perform a preliminary phytochemical analysis of the classes of constituents that are synthesized. HPLC-MS fingerprinting of the extracts from the aerial parts and roots of *P. scutellarioides* showed the presence of phenolic acids such as caffeic acid, syringic acid, ferulic acid, rosmarinic acid, chlorogenic acid, coumaric acid, and vanillic acid. In turn, cirsimaritin and eriodictiol were identified as flavonoids. Additionally, 6,11,12,14,16-pentahydroxy-3,17diacetyl-8,11,13-abietatrien-7-one, 6,11,12,14,16-pentahydroxy-3,17-diacetyl5,8,11,13-abietatetraen-7-one, and 3,6,12-trihydroxy-2-acetyl-8,12-abietadien7,11,14-trione represented the triterpenes group. These results are consistent with the literature and confirm the presence of these classes of compounds in the tested extracts of the *Plectranthus* genus. The differences in the presence of other classes of compounds in the parts analyzed is considered normal; this is due to the fact that a plant may require different compounds in its roots and aerial parts to perform similar biological functions [7,11,32,33,34,35,36].

The final step was to test the biological properties of the obtained AEPS and REPS, starting with the cytotoxic effect on the MCF-7 and A549 cancer cell lines versus the normal cell line HGF-1. Our results revealed that both extracts possessed cytotoxic effects on the tested cancer cell lines MCF-7 and A549, with different sensitivity. For the MCF-7 cancer cell line, the IC_50_ values of AEPS and REPS were 0.125 and 0.726 µg/mL, respectively, while for the A549 cell line, they were 0.850 and 0.250 µg/mL, respectively. In addition, AEPS did not show a cytotoxic effect on the HGF-1 normal cell line, while REPS in the lowest concentrations reduced the survival of these cells, but not in the concentration that was toxic to the cancer cell lines. The data in the literature show a cytotoxic effect of pure compounds isolated from the leaves of *P. scutellarioides* against the three human cancer lines tested—breast (MCF-7), pancreatic (PSN-1), and cervical (HeLa)—and the IC_50_ values obtained ranged from 17.9 μM to 29.8 μM. Low cytotoxicity against a normal lung fibroblast cell line (WI-38) was also demonstrated [33]. A similar cytotoxic effect was demonstrated on diterpenes isolated from the aerial parts of *P. scutellarioides* by Creeton et al. on human multiple myeloma stem cells and cancer cell line RPMI 8226, with IC_50_ = 11.2, 11.0, 4.5, and 9.7 μM values, respectively [37]. In turn, Bismelah et al. confirmed that the extract from the aerial parts of *P. scutellarioides* did not show a cytotoxic effect on normal HGF-1 cells, which is in line with our results [11].

In our study, we also examined the potential mechanism of apoptosis induction in MCF-7 and A549 cancer cells after treatment with AEPS and REPS. Apoptosis can be induced by various stimuli received by cells and is one of the major types of programmed cell death. An effective strategic approach in cancer therapy may be to induce apoptosis in activated cancer cells [38,39]. Due to the limited number of studies on apoptosis induction in this species, our results show, for the first time, a potential mechanism by way of altered apoptotic gene expression (*Bax*, *Bcl-2*, *TP53*, *Fas,* and *TNFSF10*), decreased mitochondrial potential, and increased ROS levels and genotoxic effect. This was also observed in morphological changes under the microscope. The results obtained in this study area showed a similar trend for both extracts tested, which may indicate similar classes of compounds and thus a similar mechanism of action. Burmistrova et al. showed that diterpen- parvifloron D, isolated from *P. ecklonii*, induced apoptosis in leukemic cells by altering mitochondrial membrane potential and cytochrome c release, enhanced by inhibiting extracellular signal-regulated kinases (ERKs) 1/2 signaling and altering ROS [40]. In contrast, Menon et al. demonstrated the induction of apoptosis in HCT-15 colon cancer cells through genotoxicity, DNA fragmentation, and caspase-3 activity using terpenoids isolated from *P. hadiensis* shoots [41]. Our previous studies on other *Plectranthus* species have shown that isolated diterpenes can induce apoptosis via the mitochondrial pathway in various cancer cells [42,43]. We suspect that these properties may be due to diterpenes, which were also found in the aerial parts and roots of *P. scutellarioides* in our current research. However, further research is needed to confirm this hypothesis.

Finally, the last step was to check for the first time the effect of AEPS and REPS on selected blood coagulation parameters. The biocompatibility tests showed that both AEPS and REPS have no negative effect on any of the coagulation processes studied, the integrity of the protein-lipid membrane of erythrocytes, or the activity of platelets. Therefore, should these components enter the bloodstream, they can be considered potentially safe. Furthermore, the slight prolongation of thrombin time (TT) observed after incubation with REPS may have a favorable effect on thrombosis prevention. However, this aspect of the bioactivity of the extract requires in-depth research to assess the influence of the compounds on the fibrin polymerization process and thrombin activity. To our knowledge, this is the first study of its kind to show biocompatibility for blood morphotic elements. Studies on the effects of *Plectranthus* extracts on blood morphotic elements were presented by Bhatt et al., who showed that an extract of *P. amboinicus* rich in phenols and flavonoids exhibited platelet aggregation capacity in an in vitro model [44]. Given the interesting results indicating the biocompatibility of the tested extracts for blood coagulation parameters, further studies are necessary to clarify their mechanisms of action in a broader experimental aspect.

In summary, this work establishes a foundation for further elucidating the biological and pharmacological potential of *P. scutellarioides* extracts. Building upon these observations with expanded in vitro testing and animal model validations could substantiate the viability of these extracts as therapeutic agents. Nevertheless, within its delimited scope, this study offers valuable preliminary evidence and direction for ongoing investigation.

## 4. Materials and Methods

### 4.1. Initiation of Plectranthus scutellarioides in In Vitro Cultures

Seeds of *P. scutellarioides* were obtained from the Botanical Garden in Brno, Czech Republic. Surface sterilization of the seeds was then carried out as follows: Approximately 200 seeds were rinsed in 200 mL of distilled water and then in 70% ethanol for 1 min. The seeds were then placed in a 15% solution of commercially available bleach (ACE) and Tween-20 (0.05% *v*/*v*) for 10 min with continuous stirring. Afterwards, the seeds were rinsed 5 times with 20 mL of distilled water. During the final rinse, they were incubated in water for 10 min. The sterilized seeds were dried on sterile filter paper sheets and then placed in Petri dishes containing agar and 1/2 MS (Murashige and Skoog) medium without sucrose. The seeds were kept at a temperature of 23 °C. Young seedlings were transferred to 300 mL Erlenmeyer flasks containing 80 mL of solid MS with 3% sucrose solidified with 0.7% agar. Root cultures were established by cutting roots from 4-week-old, in vitro-cultured *P. scutellarioides*; then, they were cultured in SH liquid medium, containing 0.2 mg/L indole-3-butyric acid (IBA). Root culture was carried out for five passages lasting 3–4 weeks each. Shoots, on the other hand, were grown on solid MS medium with 0.7% agar containing 3% sucrose and 1 mg/L 6-benzylaminopurine (BAP). The flasks were stored in a growth chamber under light conditions with a photoperiod of 18/6 h light/dark.

### 4.2. Preparation of Aerial Part and Root Extracts of Plectranthus scutellarioides

The hydromethanolic extracts were prepared from the aerial parts and roots of *P. scutellarioides* collected throughout the entire period of in vitro cultivation. The materials were lyophilized and powdered and then extracted for 15 min with an 80% (*v*/*v*) aqueous-methanol solvent (150 mL) at 35 °C using an ultrasonic bath. It was subsequently extracted two more times for 15 min each with the solvent. Then, the extracts were filtered and evaporated under reduced pressure. The obtained material was lyophilized to dryness and stored for further research [45].

### 4.3. Compositional Analyses by HPLC-ESI-QTOF-MS/MS

The aqueous extracts obtained by shaking maceration at room temperature were filtered through a nylon syringe filter (pore diameter of 0.1 µm) and subjected to HPLC-ESI-QTOF-MS/MS analysis. For this purpose, a freshly calibrated apparatus that was composed of an HPLC chromatograph 1200 Series (containing a binary pump, a degasser, an autosampler, and a thermostat) with an ESI-QTOF-MS/MS detector G6530B produced by Agilent Technologies (Santa Clara, CA, USA) was operated in both positive and negative ionization modes. The detailed chromatographic conditions were as follows: flow rate: 0.2 mL/min; temperature: 20 °C; injection volume: 10 µL; post time: 5 min; and Zorbax Eclipse Plus RP-18 chromatographic column (150 mm × 2.1. mm, 3.5 µm) used for the chromatographic separation in the following gradient of acetonitrile with 0.1% formic acid (solvent B) in 0.1% aqueous solution of formic acid (solvent A): 0 min—1% B in A, 10 min—20% of B in A, 15 min—40% of B in A, 17–18 min—95% of B in A, 19–30 min—1% of B in A. To acquire the data using the mass spectrometer, the temperature of gases was set as 250 and 300 ◦C for the carrier and sheath gas, respectively, the gas flows as 12 L/min, the capillary voltage as 3000 V, the nozzle voltage as 1000 V, the fragmentor voltage as 110 V, the skimmer voltage as 65 V, the nebulizer pressure as 35 psig, the collision energies as 10 and 20 V, and the *m*/*z* range as 40–1300 Da. A simultaneous co-injection of the calibration mixture was introduced to ensure the mass measurement was accurate. The Mass Hunter program (B.10.00) by Agilent Technologies was used to acquire and handle the obtained data. The identification of the constituents of the extracts was based on the recorded fragmentation patterns, the scientific literature, and the open mass database Metlin.

### 4.4. Cell Cultures

In the research, breast cancer cells (MCF-7) (HTB-22™; ATCC, Manassas, VA, USA), human lung cancer cells (A549) (CCL-185; ATCC, Manassas, VA, USA), and normal human gingival fibroblasts (HGF-1) (CRL-2014^TM^; ATTC, Manassas, VA, USA) were obtained from the American Type Culture Collection. The cells were cultured in MEM (MCF-7) or DMEM (A549 and HGF-1) medium supplemented with 10% fetal bovine serum (FBS), 100 µg/mL streptomycin, and 100 U/mL penicillin. All cell culture reagents were purchased from Biowest (Nuaillé, France). Cells were maintained at 37 °C at 5% CO_2_ and 100% humidity. At 70–90% confluence, cells were harvested by trypsinization; next, cells were seeded in 96-well plates at a density of 1 × 10^5^ cells per well and incubated overnight. Then, the medium was replaced with fresh medium containing the extracts.

### 4.5. Cytotoxicity Effect

The MTT test assesses cytotoxic effects and analyses cell metabolic activity. It utilizes the ability of mitochondrial dehydrogenase enzyme to convert a water-soluble tetrazolium salt into an insoluble formazan. This results in a color change from orange to dark blue. MCF-7, A549, and HGF-1 were seeded in triplicate on three 96-well microplates at a ratio of 1 × 10^5^ cells per 100 µL. They were then incubated with the prepared AEPS and REPS for 24 h at specified concentrations (0–4 mg/mL). In the next step, the medium was removed, and the cells were incubated for 4 h with 100 µL of MTT solution (5 mg/mL MTT in phosphate-buffered saline (PBS)). After this time, the plates were read in a microplate spectrophotometer (Synergy HT, BIO-TEK, Winooski, VT, USA) at 570 nm. The determined IC_50_ value indicates cell viability, providing information about the concentration at which 50% of the cells remained alive compared to the control sample. The analysis was performed in triplicate for each concentration on each of the three prepared microplates [46,47].

### 4.6. Microscopic Observation

Morphological changes induced by AEPS and REPS in MCF-7 and A549 were observed using an inverted microscope with phase-contrast. The observation was conducted following a procedure developed based on an article by Moongkarndi et al., with minor modifications [48]. Cells (5 × 10^5^) were incubated for 24 h with AEPS and REPS at a concentration corresponding to the previously determined IC_50_ in the MTT test. The media were removed, and the cells were rinsed once with PBS. Morphological changes in the cells were observed at 200× magnification using an inverted phase-contrast microscope (Leica DM IL LED Fluo; Leica Microsystems, Wetzlar, Germany).

### 4.7. Isolation of RNA from Cell Lines and Transcription into cDNA

RNA isolation was performed using A549 and MCF-7 cancer cell lines treated with AEPS and REPS at the IC_50_ concentration and untreated cells. Total RNA isolation was carried out according to the manufacturer’s instructions using the Total RNA MIDI kit (A&A Biotechnology, Gdansk, Poland). The RNA content was assessed using the Biotek Synergy HT Microplate Reader. Subsequently, the obtained RNA was transcribed into cDNA using the TranScriba kit (A&A Biotechnology, Gdansk, Poland), following the provided instructions.

### 4.8. Gene Expression

MCF-7 and A549 cancer cells were incubated with AEPS and REPS at an IC_50_ concentration for 24 h. Gene expression was analyzed using TaqMan Probe-Based Real-Time PCR. TaqMan probes (Life Technologies, Carlsbad, CA, USA) were used to analyze five genes with the following probes: (Hs00608023_m1 (BCL2), Hs00180269_m1 (BAX), Hs00153349_m1 (TP53), Hs00236330_m1 (Fas), and Hs00921974_m1 (TNFSF10), and the reference gene was the 18S rRNA gene (Hs99999901_s1) (Life Technologies, Carlsbad, CA, USA). The RT-qPCR was carried out using the TaqMan™ Gene Expression Master Mix (ThermoFisher Scientific, Waltham, MA, USA) and the Agilent Technologies Stratagene Mx300SP (Agilent Technologies, Santa Clara, CA, USA), working with MxPro software version 4.10. RT-qPCR conditions included 10 min polymerase activation at 95 °C, followed by 40 cycles of 30 s denaturation at 95 °C and 60 s annealing/extension at 60 °C. Samples were run in triplicate. The basal level of expression was calculated according to the Ct method [49].

### 4.9. Genotoxicity Assay (Comet Assay)

The genotoxicity of AEPS and REPS was determined using the comet assay. The final concentration of MCF-7 and A549 cancer cells in each sample was adjusted to 1 × 10^5^ cells/mL. The following IC_50_ concentrations of AEPS and REPS were used. As previously described [50], the comet assay was performed under alkaline conditions (pH > 13). Cells were incubated with both extracts for 1 h at 37 °C. Samples were centrifuged (182× *g*, 15 min, 4 °C). Cells were resuspended in 0.75% L-MMP agarose, layered on slides precoated with 0.5% N-MMP agarose, and lysed for 1 h at 4 °C in a buffer consisting of 2.5 M NaCl, 1% Triton X-100, 100 mM EDTA, and 10 mM Tris, pH 10. After lysis, to allow the DNA to unwind, electrophoresis was performed in an electrophoresis solution containing 300 mM NaOH and 1 mM EDTA. The electrophoresis was carried out for 20 min under an electric field strength of 0.73 V/cm (300 mA). Slides were neutralized, air-dried overnight, and stained with 1 µg/mL DAPI. The comets were observed at a magnification of 200× using a fluorescence microscope (Nikon Eclipse C. H600L, Tokyo, Japan) connected to a video camera and a personal computer-based image analysis system (Lucia-Comet v. 7.0, Laboratory Imaging, Prague, Czech Republic). Fifty images per sample were randomly selected for analysis. DNA damage was measured as the percentage of DNA in the comet tails. Two parallel assays were performed on aliquots from the same sample. The total number of cells was 100, and the mean DNA damage was calculated.

### 4.10. Intracellular ROS Measurement

Dichlorofluorescein diacetate (H2DCFDA, Invitrogen™, Waltham, MA, USA), a membrane-permeant fluorescent probe widely used to monitor intracellular ROS production, was used to analyze intracellular ROS generation [51]. The MCF-7 and A549 cancer cells used for analysis were seeded in 96-well plates at 1 × 10^5^ cells/well in 50 μL culture medium and cultured at 37 °C for 12 h in an environment containing 5% CO_2_. The cells were then treated with AEPS and REPS at IC_50_ concentrations and incubated for 1 h to 24 h. Untreated cells served as controls. After treatment, the cells were incubated with 5 µM DCFH-DA (prepared in HBSS buffer) at 37 °C for 45 min. Fluorescence was measured using a Bio-Tek Synergy HT Microplate Reader (Bio-Tek Instruments, Winooski, VT, USA) at an excitation wavelength of 480 nm and an emission wavelength of 510 nm. The intensity of DCF fluorescence was expressed.

### 4.11. Assay for the Mitochondrial Membrane Potential (JC-1 Assay)

Mitochondrial membrane potential (MMP) was determined using the mitochondria-specific cationic fluorescent dye JC-1. JC-1 (5,5′,6,6′-tetrachloro-1,1′3,3′-tetraethylbenzamidazol-carbocyanine iodide) is a lipophilic dye that accumulates in mitochondria as a function of potential. It is a dual-emission fluorescent dye that can selectively enter mitochondria. When the membrane potential is lowered, it can reversibly change color from red to green. JC-1 aggregates and emits intense red fluorescence in healthy cells with normally polarized mitochondria. However, in unhealthy or apoptotic cells with depolarized mitochondrial membranes, it forms monomers that give off green fluorescence. Depolarizing MMPs is one of the key early events of apoptosis [42,52]. MCF-7 and A549 cancer cells were seeded in black 96-well tissue culture plates with a transparent bottom (Greiner Bio-One, Monroe, NC, USA) at a density of 1 × 10^5^ cells/well in 50 µL culture medium. The cells were allowed to grow overnight. The next day, the cells were treated with the indicated concentration (IC_50_) of AEPS and REPS for 24 h. Finally, cells were preincubated with 5 μM JC-1 in HBSS in a CO2 incubator at 37 °C for 30 min. Before measurements, cells were centrifuged (300× *g* for 10 min at 22 °C) and washed twice with HBSS. Fluorescence was measured using a Bio-Tek Synergy HT Microplate Reader. The filter pairs used were 530 nm/590 nm and 485 nm/538 nm. Triplicates were used to calculate the percentage of cells showing MMP changes. Results were expressed as the ratio of red to green fluorescence in comparison to the control.

### 4.12. Biocompatibility Studies

The preliminary study of biocompatibility was performed using human blood incubated with various concentrations of the tested extracts. The impact on three basic laboratory tests of plasma coagulation (PT, APTT, TT) and interactions with the erythrocyte membrane and platelets were assessed.

#### 4.12.1. Biological Materials

Blood samples were obtained from healthy donors from the Blood Donation Centre in Lodz. The experiments on the biological material were accepted by the Bioethics Committee of the Medical University of Lodz (Medical University of Lodz, Poland, approval No. RNN/105/20/KE). The studies on human biological material were conducted in accordance with Polish national directives. The tested human blood constituted a residual material of routine diagnostic studies destined for removal as medical waste. The blood was collected into vacuum tubes filled with 3.2% buffered sodium citrate. The preparation of biological materials was described previously [53].

#### 4.12.2. Basic Coagulation Tests: PT, APTT, and TT

The measurements of PT, APTT, and TT were conducted using a coagulometer (CoagChrom-3003 Bio-Ksel, Grudziadz, Poland) according to the routine procedure, using commercially available reagents obtained from Bio-Ksel, Poland. In every test, control samples consisting of methanol diluted with distilled water (1:5) were included. The experiment was conducted as an independent experiment in duplicate (*n* = 4–5). The results are presented as mean ± standard deviation (SD).

#### 4.12.3. Erythrotoxicity

The influence of extracts on red blood cells’ (RBCs) membrane integrity was performed by lysis assay, which was conducted as previously described [53].

Briefly, 2% RBC suspension in NaCl was incubated at 37 °C for 24 h with the tested extracts at concentrations ranging from 1 to 100 μg/mL or methanol in control samples. Afterwards, the samples were centrifuged at 3000× *g* for 10 min, and the absorbance of the supernatant was collected at 550 nm. The results were presented as the degree of hemolysis, which constituted a percentage of released hemoglobin. A sample containing 10 μL of 2.0% *v*/*v* Triton X-100 was used as a positive control (100% of hemolysis), whereas a sample of saline solution represented spontaneous hemolysis of RBCs (control). The experiments were conducted using at least four different biological materials (*n* = 3). The results are presented as mean ± standard deviation (SD).

#### 4.12.4. Red Blood Cell Morphology

A 2% RBCs suspension was incubated at 37 °C for 24 h with various concentrations of tested extracts. Afterwards, erythrocyte morphology was evaluated using a phase contrast Opta-Tech inverted microscope, at 400-times magnification, equipped with (OptaView 7) for image analysis.

#### 4.12.5. Total Thrombus-Formation Analysis System (T-TAS)

For quantitative analysis of white thrombus formation T-TAS^®^ (Total Thrombus formation Analysis System), an AR-Chip was used (ZACROS, Japan http://www.t-tas.info, accessed on 1 December 2023). T-TAS^®^ is based on technology using a flow chamber system that can quantitatively measure the thrombus formation process under physiological flow conditions. The AR-Chip is pre-coated with type I collagen (pig-derived) and tissue thromboplastin (rabbit-derived) on its surface for the flow paths. Citrated blood treated with CaCTI reagent (contains CTI (corn-derived trypsin inhibitor, the inhibitor for FXII) and CaCl_2_) is applied into the AR chip flow path, and platelets are activated by the effect of collagen and 600 s^−1^ wall shear stress. Furthermore, the blood coagulation pathway is activated by tissue thromboplastin and calcium ions. In this mechanism, the intrinsic coagulation pathway is eliminated because of inhibition of FXII by the effect of CTI. Thrombi are formed as a result of primary and secondary (especially with the extrinsic pathway) hemostasis, and the pressure inside the flow path increases due to the occlusion of the flow paths. Blood (495 μL) was incubated with 5 µL of tested extracts at the final concentrations 1, 10, and 100 μg/mL (5 min, 37 °C). The results were recorded as changes of flow pressure in time. The evaluated parameters were as follows: AUC30—area under the flow pressure curve recorded for 30 min after the start of the test; OST—occlusion start time (the lag time for the flow pressure to reach 10 kPa due to partial occlusion of the capillary); OT—occlusion time (the lag time for the flow pressure to reach 60 kPa from baseline pressure). The results are presented as mean ± SD. The blood samples were collected from 4 healthy volunteers.

### 4.13. Statistical Analysis

Data are presented as mean values with standard deviation (mean ± SD). One-way analysis of variance followed by Tukey’s post hoc test was used for statistical comparisons. A statistically significant difference was considered as *p* < 0.05.

## 5. Conclusions

In conclusion, this study shows that in vitro cultures of *Plectranthus scutellarioides* can be an alternative to wild plants as well as an easily accessible source of valuable secondary metabolites, exhibiting a number of biological properties. Extracts from the aerial parts and roots showed the presence of such classes of compounds as phenols, flavonoids, and terpenoids, as well as cytotoxic activity against MCF-7 breast and A549 lung cancer cells. Both extracts induced cell death mainly through apoptosis, as a result of changing the regulation of the expression of several genes, such as *Bax*, *TP53*, *Bcl-2*, *Fas,* and *TNFSF10*, decreased mitochondrial membrane potential, increased ROS, and increased DNA damage. The extracts also showed moderate blood coagulation properties. The results obtained suggest that extracts from in vitro cultures of *Plectranthus scutellarioides* contain many valuable classes of bioactive compounds, which may be responsible for the biological properties analyzed. However, further studies are needed to explain the mechanisms of action so that in the future we can use these species in the treatment of many diseases.

## Figures and Tables

**Table 1:** The list of tentatively identified components of the investigated aerial parts and roots of *P. scutellarioides* extracts by HPLC-ESI-QTOF-MS/MS.

No.	Ion.+/−	Rt (min)	Molecular Formula	*m*/*z* Theoretical	*m*/*z* Experimental	Error	DBE	MS/MS Spectrum	Proposed Compound	Root	Aerial Part
1	-	6.1	C_15_H_12_O_6_	287.0561	287.0565	−1.35	10	ND	Eriodyctiol	++	++
2	-	7.5	C_8_H_14_O_4_	173.0455	173.0468	−7.2	3	155, 130, 111	Propylglutaric acid	+++	+++
3	-	11.5	C_24_H_32_O_10_	479.1923	479.1952	−6.1	9	ND	6,11,12,14,16-Pentahydroxy-3,17diacetyl-8,11,13-abietatrien-7-one	++	++
4	-	12.2	C_8_H_8_O_4_	167.035	167.0363	−7.84	5	149, 123, 121, 109	Vanillic acid	+++	+++
5	-	12.3	C_24_H_30_O_10_	477.1766	477.1732	7.15	10	ND	6,11,12,14,16-Pentahydroxy-3,17-diacetyl5,8,11,13-abietatetraen-7-one	++	++
6	-	12.3	C_9_H_10_O_5_	197.0455	197.0470	−7.34	5	179, 135, 123	Syringic acid	+++	+++
7	-	12.9	C_16_H_18_O_9_	353.0878	353.0903	−7.04	8	ND	Chlorogenic acid	++	+++
8	-	13.1	C_7_H_6_O_4_	153.0193	153.0200	−4.34	5	123, 109	Dihydroxybenzoic acid	+++	+++
9	-	13.7	C_22_H_28_O_8_	419.1711	419.1673	9.14	9	ND	3,6,12-Trihydroxy-2-acetyl-8,12-abietadien7,11,14-trione	+	+
10	-	15.1	C_7_H_6_O_3_	137.0244	137.0245	−0.6	5	108	Hydroxybenzoic acid	++	+++
11	-	17.2	C_18_H_16_O_8_	359.0772	359.0780	−2.11	11	179, 135	Rosmarinic acid isomer	+	+++
12	-	17.8	C_9_H_8_O_4_	179.0350	179.0361	−9.21	6	135	Caffeic acid	++	+++
13	-	19.3	C_9_H_8_O_3_	163.0401	163.0413	−7.51	6	119, 108	Coumaric acid	+	++
14	-	20.9	C_18_H_16_O_8_	359.0772	359.0788	−433	11	197, 161, 135	Rosmarinic acid	++	+++
15	-	21.1	C_10_H_10_O_4_	193.0506	193.0492	7.38	6	178, 161, 134	Ferulic acid	+++	+++
16	-	22.3	C_17_H_14_O_6_	313.0718	313.0708	3.06	11	293, 194, 161	Cirsimaritin	+++	+++

Rt—retention time, DBE—double bond and ring number, error—error of *m*/*z* measurement, +—peak area of 1 × 10^5^–1 × 10^6^, ++—peak area of 1 × 10^6^–1 × 10^7^, +++—peak area higher than 1 × 10^7^, ND—no data.

**Table 2:** Effects of AEPS and REPS on the parameters of white thrombus formation process (mediated by blood platelet activation) and coagulation processes, under blood flow conditions using T-TAS (AR-chip). Whole blood samples were analyzed at the shear stress rates of 600 s^−1^ (10 μL/min).

		OST—Occlusion Start Time (s)		OT—Occlusion Time (s) **		AUC—Area under the Curve **	
		*REPS*	*AEPS*	*REPS*	*AEPS*	*REPS*	*AEPS*
CTR		402 ± 16.9	335 ± 8.5	466 ± 5.7	399 ± 3.5	1373 ± 5.7	1455 ± 30.4
1 µg/mL		421 ± 10.7	292 ± 15.3	464 ± 10.6	443 ± 7.9	1365 ± 6.4	1389 ± 53.4
10 µg/mL		405 ± 24.0	302 ± 3.5	452 ± 27.6	408 ± 16.2	1380 ± 26.9	1389 ± 53.4
100 µg/mL		408 ± 55.2	360 ± 8.8	472 ± 43.8	467 ± 15.2	1371 ± 48.1	1369 ± 45.2
interpretation	low risk of adverse events	*		357–729 s		1257–1422	
	high risk of bleeding	*		>729 s		<1257	
	high risk of thromboembolic events	*		<357 s		>1422	

* no literature data available. ** http://www.t-tas.info, accessed on 1 December 2023.

## Data Availability

The data presented in this study are available on request from the corresponding author.

## Supplementary Materials

The supporting information can be downloaded at: https://www.mdpi.com/article/10.3390/ijms25021043/s1.
