# Supplementary material for: Biological Properties of Extracts Obtained from In Vitro Culture of Plectranthus scutellarioides in a Cell Model
Source: Int J Mol Sci. 2024 Jan 15;25(2):1043. doi: 10.3390/ijms25021043 (PMC10815897; doi:10.3390/ijms25021043)
Supplement: Supplementary file 1 [file ijms-25-01043-s001.zip › ijms-2806340-supplementary.pdf]

SUPPLEMENTARY FILE

Table S1. Mass chromatograms of the tested samples recorded in negative and positive ionization modes

Type of mass chromatogram	Chromatogram
Mass chromatogram from the aerial parts in the negative ionization mode	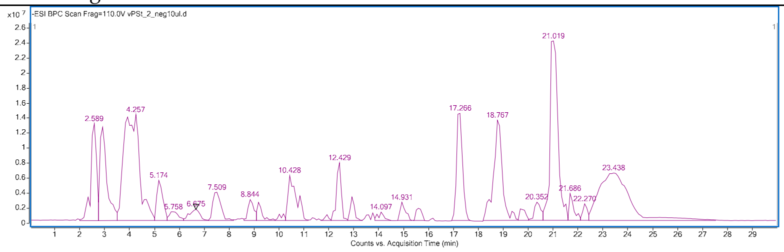
Mass chromatogram from the aerial parts in the positive ionization mode	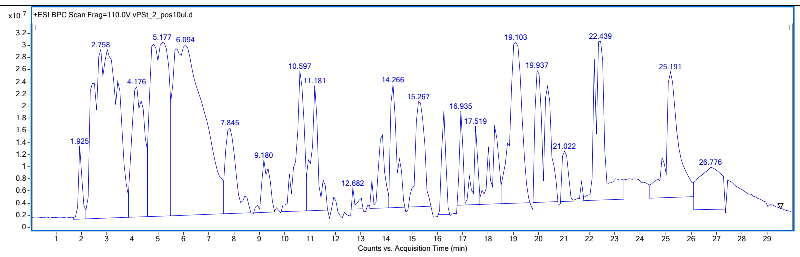
Mass chromatogram from the roots in the negative ionization mode	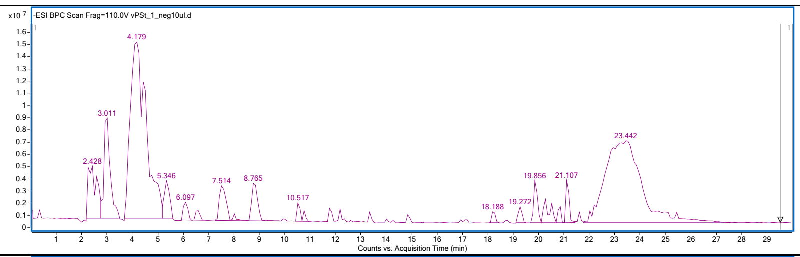
Mass chromatogram from the root in the positive ionization mode	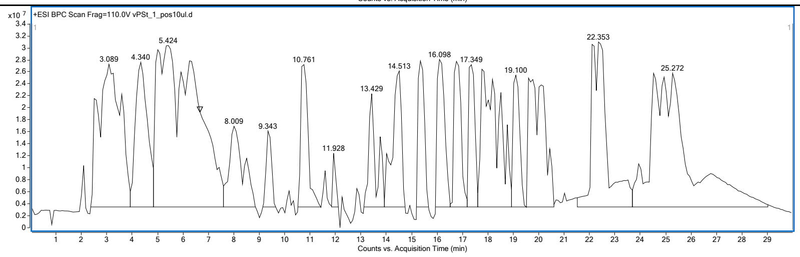

Table S2. The list of MS/MS spectra of the tentatively identified compounds

No	MS/MS spectrum	Proposed compound
**1**	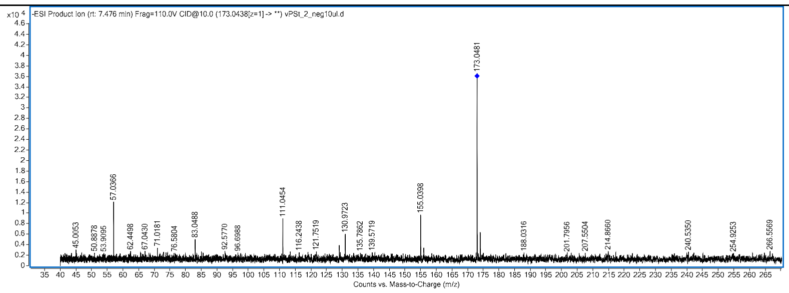	Propylglutaric acid

2	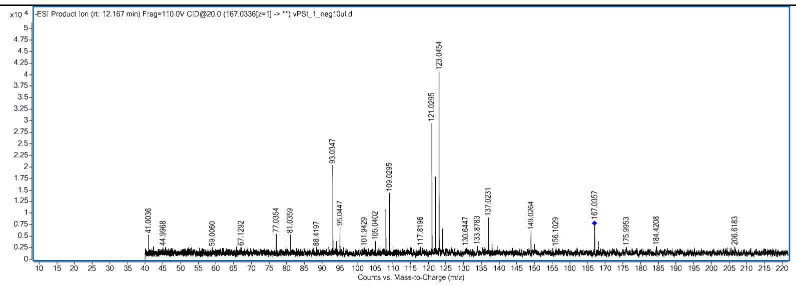	Vanillic acid
3	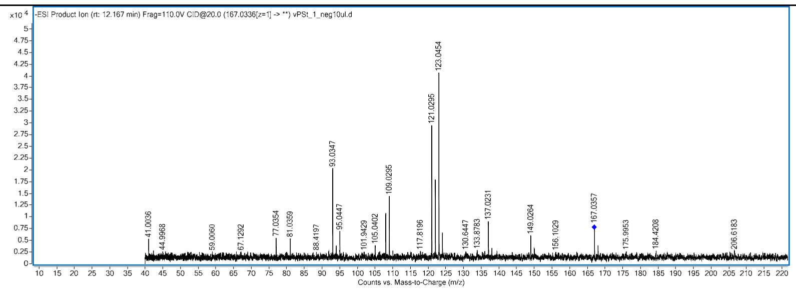	Syringic acid
4	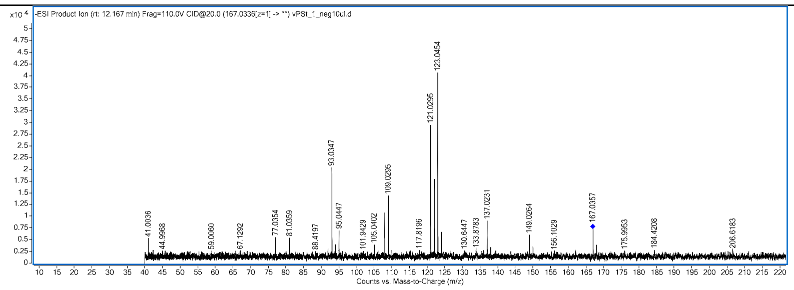	Dihydroxybenzoic acid
5	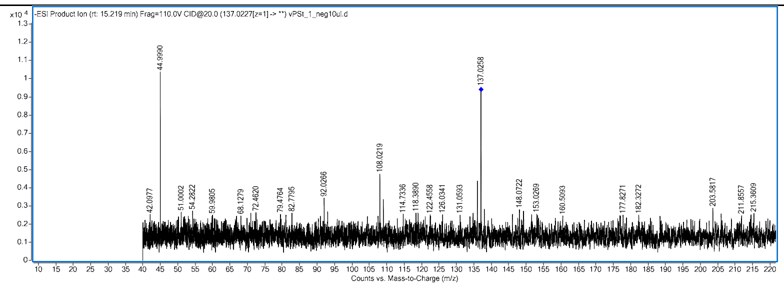	Hydroxybenzoic acid
6	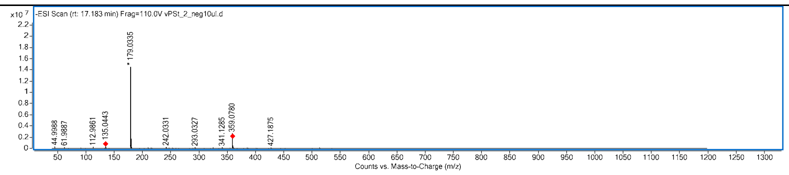	Rosmarinic acid isomer
7	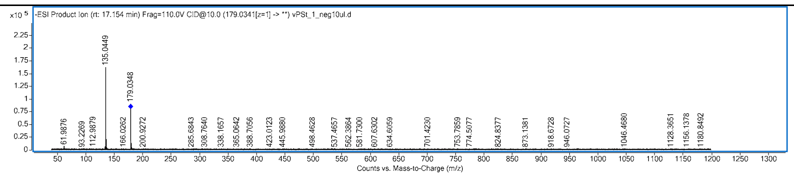	Caffeic acid
8	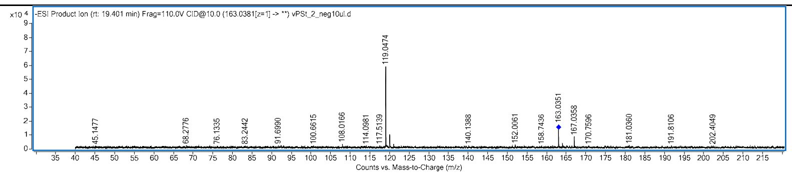	Coumaric acid
9		Ferulic acid

	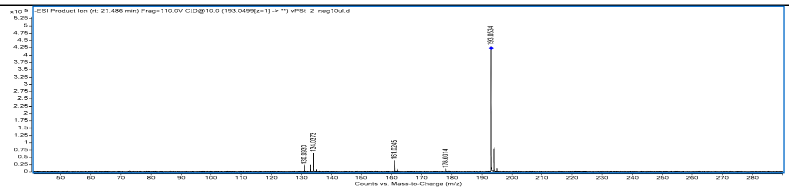	
**10**	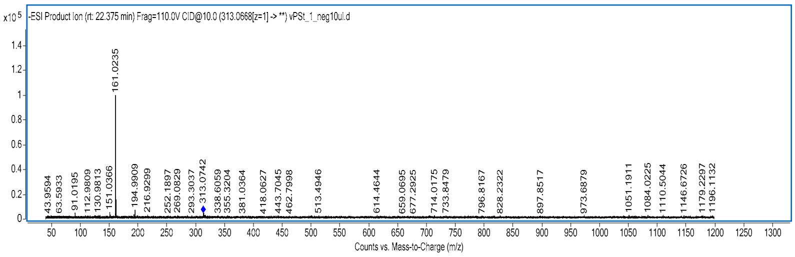	Crisimaritin
**11**	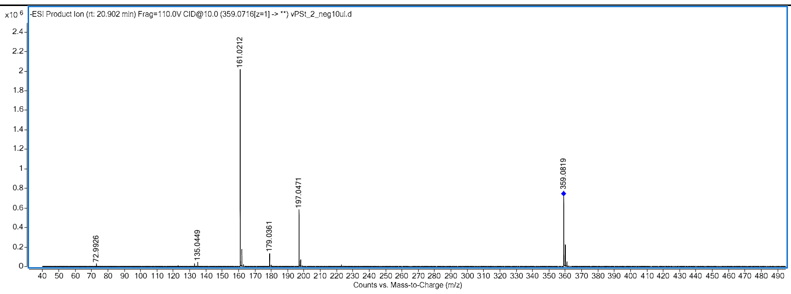	Rosmarinic acid
